# Influence of inflammation on bleeding and wound healing following surgical extraction of impacted lower third molars

**DOI:** 10.1186/s12903-023-02754-0

**Published:** 2023-02-09

**Authors:** Yusheng Cheng, Maged Ali Al-Aroomi, Naseem Ali Al-Worafi, Essam Ahmed Al‑Moraissi, Changfu Sun

**Affiliations:** 1 Department of Stomatology, First People’s Hospital of Xuzhou, Xuzhou Municipal Hospital Affiliated of Xuzhou Medical University, Xuzhou, Jiangsu People’s Republic of China; 2 Department of Oroamxillofacial-Head and Neck Surgery, School of Stomatology, China Medical University, No.117. Nanjing North Street, Heping District, Shenyang, 110002 Liaoning People’s Republic of China; 3 Department of Orthodontics, School of Stomatology, China Medical University, Shenyang, People’s Republic of China; 4 Department of Oral and Maxillofacial Surgery, Faculty of Dentistry, Thamar University, Thamar, Yemen

**Keywords:** Third molars, Surgical extraction, Bleeding, Wound healing

## Abstract

**Objective:**

This study aimed to investigate the effect of inflammatory states following impacted lower third molar (ILTM) surgery regarding postoperative bleeding and wound healing.

**Methods:**

The study included patients who underwent extraction of ILTMs associated with or without inflammatory conditions. Post-extraction bleeding and wound healing were assessed. In addition, mean grey values (MGVs) of alveolar bone and bone height using an orthopantomography radiograph were analyzed.

**Results:**

A total of 376 patients were enrolled; 171 pericoronitis, 51 pulpitis, 44 chronic periapical periodontitis, 36 chronic periodontitis, and 74 control. The bleeding score in the control group was significantly lower than in the periapical periodontitis and periodontitis groups. Excellent wound healing for control, pericoronitis, pulpitis, periapical periodontitis, and periodontitis groups was (78.38%, 35.67%, 70.59%, 70.45%, and 33.33%, respectively). Patients with pericoronitis and periodontitis had significantly poorer wound healing (*P* < 0.01). The MGV in periapical periodontitis and periodontitis was considerably lower than in the control group.

**Conclusions:**

The inflammatory conditions associated with ILTMs increase the risk of bleeding. So suturing with the placement of local hemostatic agents over a pressure pack alone is recommended. The poorest wound healing was in localized gingival inflammation. Furthermore, MGV was affected by age and was lower with periapical periodontitis.

## Introduction

Extraction of impacted teeth is one of the most common invasive oral surgical procedures in routine dental practice [1]. Impacted lower third molar (ILTM) are found in 20–30% of the population, with a higher prevalence in females due to the smaller jaws [2, 3]. The late formation of third molars and the evolution of the size of the mandible resulted in insufficient space for proper eruption. As a result, impacted third molars are frequently extracted as a preventative intervention [4]. However, there is always a debate on whether to retain or extract an asymptomatic ILTM.

Pericoronitis, pulpitis, periapical lesion, periodontitis, bone loss, neoplasms, and root resorption of the adjacent teeth are potential for pathologic sequelae associated with ILTM, as well as one of the main reasons for their extraction [5–7]. Surgical extraction of ILTM, like any surgical procedure, could be associated with postoperative complications such as swelling, severe pain, sensory nerve damage, trismus, infection, and dry socket [8]. Adequate surgical methods, such as selecting an appropriate flap design, minimal bone removal, and less trauma to adjacent soft tissues with proper wound closure techniques could decrease the incidence of postoperative sequelae but not eliminate it [9].

Local inflammation has varying degrees of influence on the effect of local anesthesia during the extraction of ILTM, control of intraoperative and postoperative bleeding, and the healing of extraction wounds, which result in discomfort and significant morbidity [10]. Therefore, surgeons have a great interest in minimizing these complications to improve patient satisfaction and reduce additional follow-up visits.

Based on the author's knowledge, there was no study comparing the impact of the presence of local inflammatory conditions with ILTMs to healthy asymptomatic ILTM surgeries in respect of postoperative wound healing and bleeding. Thus, this study aims to compare between different concurrent inflammatory statuses with ILTM surgery regarding postoperative complications such as bleeding and wound healing.

## Material and methods

### Study design

The study protocol and informed consent form were reviewed and approved by the ethical and research committee at the school of stomatology, China medical university, and has been conducted in full accordance with the declaration of Helsinki, and patients signed the informed consent before sample collection. This prospective parallel study included patients who underwent unilateral surgical extraction of ILTM surgery. Based on history, clinical and radiographical examination, the study group (302 patients) was further categorized into four subgroups; chronic pericoronitis group, acute pulpitis group, chronic periapical periodontitis group, and chronic periodontitis group (no overlaps between those subgroups). The control group included asymptomatic patients without any associated pathology or local inflammatory conditions (74 patients).

### Inclusion criteria

(1) Age ranged between 18 and 55 years, (2) Generally healthy (no blood disorders or history of any medications) with no psychological or mental history, (3) Pell and Gregory [11] class II, position B impacted teeth with the same surgical difficulty, (4) Homogenous of horizontally impacted teeth (to ensure that all patients were a similar at the baseline in respect of surgical difficulties, thus, eliminating any confounding factors that could effect on the result).

### Exclusion criteria

Patients under 18 years of age, preoperative removal of an adjacent second molar, pregnancy and lactation, patients with a history of the irradiated maxillofacial region, and immunocompromised patients with diabetes or hypertension were excluded from the study. Also, patients with bleeding disorders were excluded.

### Surgical procedure

All surgery was performed using identical surgical instruments and material, and one highly experienced surgeon performed all surgeries. Before surgery, all participants underwent a radiological examination, including an orthopantomography radiograph (OPG). In all cases, the inferior alveolar, lingual, and buccal nerves were anesthetized using 1.7–3.4 mL of 2% Articaine hydrochloride and epinephrine anesthetic solution at 1:100,000 (1 or 2 carpules). The extractions were accomplished by elevating a full-thickness mucoperiosteal flap with releasing incision on the distobuccal aspect of the second molar. Following the reflection of the flap in the conventional dentoalveolar surgery, osteotomy was performed with a straight hand-piece under continuous irrigation with normal saline—tooth sectioning and gently elevated. Regional infiltration anesthesia will be used in severe or moderate pain during the extraction. After tooth removal, the surgical field was rinsed with sterile 0.9% saline. The extractions sites were closed using 3–0 silk material (as it was the only material available in the hospital at that time) with the interrupted suturing method. After surgical extraction, all patients received an intra-oral gauze pressure pack and were reviewed after 30 min to confirm hemostasis. The usual postoperative instructions were given to the patients—the suture removal on day 7 after the operation.

### Postoperative observations

#### Postoperative bleeding

Post-extraction bleeding was assessed on a five-point scale, with grade 0 indicating no bleeding; grade I indicating very mild bleeding; grade II indicating mild bleeding; grade III indicating moderate bleeding, and grade IV indicating severe bleeding [12].

The gauze pack was removed 30 min after the extraction; if hemostasis was satisfactory (grade 0), the patient was then discharged from the clinic. If oozing persists (grade II or above), intra-oral pressure packs were again applied, and sockets were reexamined after 30 min. The sockets were reexamined, and if oozing was observed, different treatment procedures were used regarding the bleeding score (grade II; tightly sutured with bite firmly, grade III; a hemostatic agent was packed into the surgical site, grade IV; used bipolar cautery and hemostatic agent packed into the surgical site).

#### Postoperative wound healing

At the 7th postoperative day, the patients were called back for suture removal and postoperative soft tissue healing assessment. Wound healing was assessed according to the Landry and Turnbull criteria [13] on a four-point scale, with grade 0 indicating excellent healing (tissues are pink without swelling); grade I indicating good healing (tissues slight redness and swelling about 25–50%); grade II indicating poor wound healing (tissues obvious redness, swelling with bleeding, more than 50% with connective tissue exposed); grade III indicating very poor wound healing (loss of epithelium beyond margins with infection).

#### Bone density

**Fig. 1:**
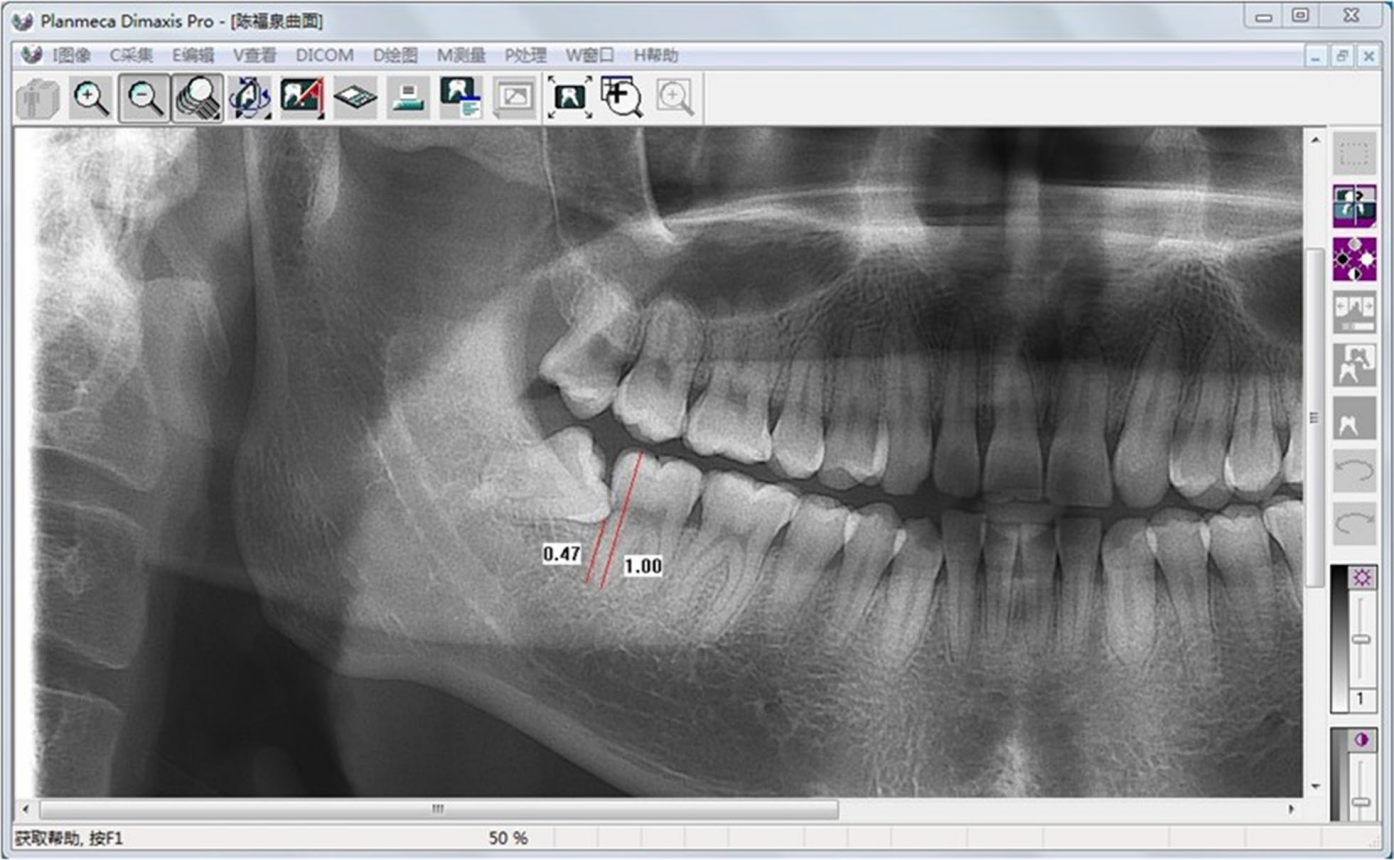
Perioperative radiograph patient shows the site for measuring bone height

Three months post-extraction, the mean grey values (MGVs) of newborn alveolar bone were analyzed using OPG with Planmeca Dimaxis Pro 4.5 software (PLANMECA co., Finland). A single observer performed all screening procedures, and the radiographic readings depended on the observer's reading skill. The image was adjusted, magnified, and automatically focused just at the ILTM alveolar socket to analyze the regions of interest. The upper border is the highest point of the socket coronally, and the lower border is the lowest. At the same time, the MGV of the second molar crown was measured as a reference and used to evaluate the bone mineral density of the extracted socket. The bone height was determined as the distance from the second molar distal root, and the mesial wall of the extraction socket was contacted. A straight line was drawn parallel to the second molar long axis, from the most apical point in the root apex to the highest contact point coronally (Fig. 1).

### Statistical analysis

The statistical analysis was performed using SPSS version 19 (Windows, IBM). The demographic data were analyzed using *x*^*2*^ test. Paired t-test and Wilcoxon test were used to determine whether there were significant differences among the groups.

## Results

A total of 376 patients (205 men and 171 women) met the inclusion criteria with an age range between 18 and 55 years. The majority of impactions (45.48%, n = 171) were chronic pericoronitis, followed by (19.68%, n = 74) healthy asymptomatic (control group), (13.56%, n = 51) chronic periapical periodontitis, (13.56%, n = 51) acute pulpitis, and (9.58%, n = 36) chronic periodontitis. The descriptive data for the sample are summarized in Table 1.

**Table 1:** Demographic data of the patients and characteristics

Age	Control (Normal)		Pericoronitis		Pulpitis		Periapical periodontitis		Periodontitis	
	M	F	M	F	M	F	M	F	M	F
18–25	8	10	25	33	8	6	4	2	3	1
26–35	12	8	29	37	11	8	6	5	1	0
36–45	13	9	17	13	5	3	8	6	9	4
46–55	8	6	10	7	6	4	9	4	13	5
Total	74		171		51		44		36	
	376									

**Fig. 2:**
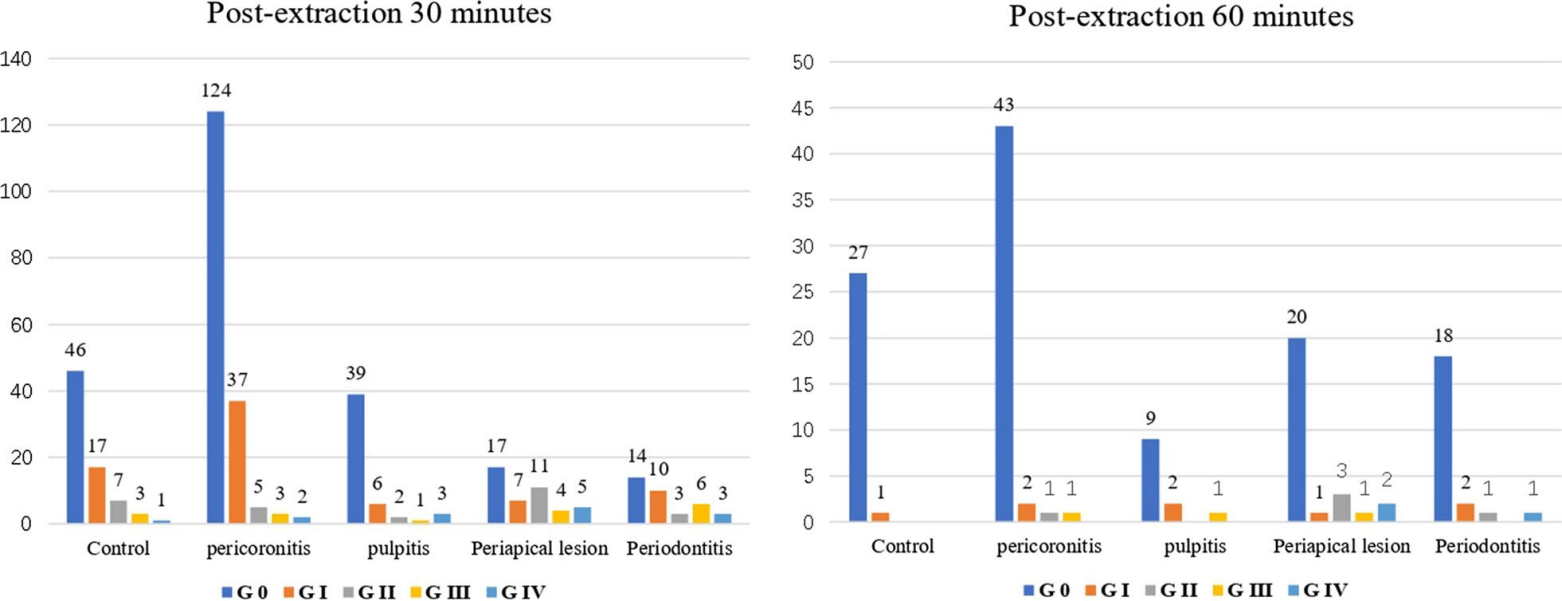
Comparison of post-extraction bleeding scores among study groups

Figure 2 shows the score of postoperative bleeding after tooth extraction. On the 60 min after the extraction, Grade 0 was reported in 96.43% (27/28) for control group; 91.49% (43/47) for pericoronitis group; 75% (9/12) for pulpitis group; 74% (20/27) for periapical periodontitis group; and 81.82% (18/22) for periodontitis group. Wilcoxon test showed that the bleeding score in the normal group was statistically significantly lower compared with periapical periodontitis and periodontitis groups (*P* = 0.001, *P* = 0.007, respectively). No significant differences were observed between periodontitis and pulpitis groups compared to control group (*P* = 0.064, *P* = 0.147, respectively) (Fig. 2).

**Fig. 3:**
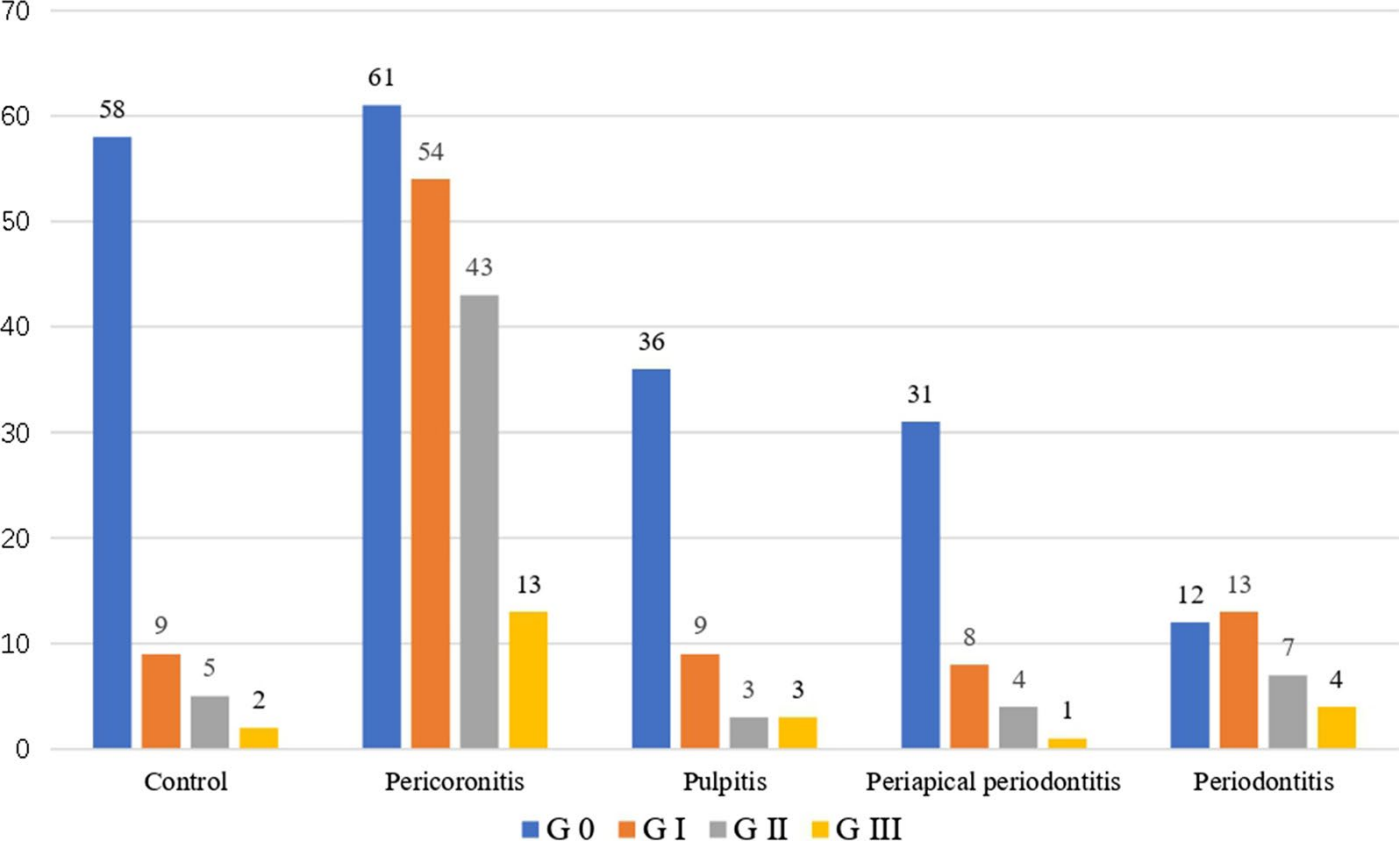
Comparison of the score of wound healing after extraction between groups

On 7 postoperative days, the excellent wound healing score for control, pericoronitis, pulpitis, periapical periodontitis, and periodontitis groups was (78.38%, 35.67%, 70.59%, 70.45%, 33.33%, respectively) (Fig. 3). Compared to the control group, the Wilcoxon test showed patients with pericoronitis and periodontitis groups had significantly poor wound healing (*P* < 0.01 for both). In contrast, there was no significant difference for pulpitis and periapical periodontitis groups (*P* = 0.175, *P* = 0.205, respectively).

Three months postoperatively, MGV of the extracted socket and alveolar bone height were analyzed for 86 patients who completed the follow-up period. The differences between the alveolar bone formation according to the assessment period experienced by study groups are summarized in Table 2. The MGV of the extracted socket in periapical periodontitis and periodontitis groups was lower than that in the control group (0.583 ± 0.081, 0.411 ± 0.103, 0.712 ± 0.092, respectively), (*P* < 0.05, Paired t-test). Table 2 also shows that elderly patients 46–55 years tend to have lower MGV than young patients18-25 years (0.397 ± 0.108, 0.687 ± 0.103, respectively) (*P* < 0.05, Paired t-test). Although, there was no significant difference of hemostasis interventions for post-extraction bleeding events for all groups regarding MGV (*P* > 0.05) (Table 2).

**Table 2:** Alveolar bone assessment experienced by study groups at 3 months after extraction

Variable	Mean score (standard deviation SD)				
	No	Mean grey values	Perioperative bone height	Postoperative bone height	Bone height difference
*Study groups*					
Control	25	0.712 ± 0.092	0.549 ± 0.074	0.447 ± 0.079	0.063 ± 0.051
Pericoronitis	17	0.652 ± 0.033	0.733 ± 0.062	0.671 ± 0.058	0.067 ± 0.044
Pulpitis	19	0.592 ± 0.074	0.622 ± 0.084	0.537 ± 0.066	0.065 ± 0.057
Periapical periodontitis	14	0.583 ± 0.081	0.546 ± 0.075	0.496 ± 0.093	0.075 ± 0.062
Periodontitis	11	0.411 ± 0.103	0.499 ± 0.134	0.488 ± 0.168	0.083 ± 0.096
*Aged group*					
18–25	28	0.687 ± 0.103	0.553 ± 0.034	0.478 ± 0.076	0.069 ± 0.083
26–35	23	0.433 ± 0.067	0.513 ± 0.052	0.449 ± 0.081	0.075 ± 0.094
36–45	20	0.632 ± 0.094	0.479 ± 0.057	0.418 ± 0.092	0.086 ± 0.079
46–55	15	0.397 ± 0.108	0.427 ± 0.093	0.395 ± 0.121	0.092 ± 0.183
*Treatment method*					
Gauze pack	73	0.662 ± 0.082	0.513 ± 0.091	0.417 ± 0.074	0.073 ± 0.069
Suturing	15	0.582 ± 0.114	0.622 ± 0.084	0.398 ± 0.104	0.068 ± 0.112
Hemostatic agent	9	0.623 ± 0.093	0.498 ± 0.127	0.428 ± 0.086	0.077 ± 0.139
Cauterized and hemostatic agent	6	0.586 ± 0.167	0.474 ± 0.144	0.501 ± 0.106	0.062 ± 0.158

Similarly, there was some bone loss regarding the alveolar ridge height distal of the second molar for all groups. It was more significant in periodontitis and elderly patient groups (0.083 ± 0.096, 0.092 ± 0.083, respectively) (Table 2).

## Discussion

Our study found that prophylactic surgical extraction of asymptomatic ILTMs is rare in our environment except for orthodontic considerations. ILTM is often considered a troublemaker and functionally nonessential, thus extracted most frequently [14]. Complications after ILTM surgical extraction are related to various factors, including surgical, tooth status, and patient factors [15]. Patients' associated factors such as anxiety, fear, and the influence of other systemic diseases, were excluded from this study.

The presence of partial and/or soft tissue-impacted ILTM is associated with a significantly increased risk of increased plaque accumulation and pericoronitis. Recurrent or chronic pericoronitis was the most common indication of surgical removal of ILTM in the current study. Although, our findings were consistent with other studies in the literature [16, 17].

Post-extraction bleeding is one of the treatment complications of dental extraction that might make a patient panic and seek immediate dental consultation, especially for those taking anticoagulants and hemorrhagic diseases patients. Although, postoperative bleeding can also occur due to local or systemic problems not expected in routine dental extractions [18]. The postoperative bleeding rate for mandibular and maxillary third molar extraction was 0.6% and 0.4%, respectively [19]. Many authors believe that post-extraction bleeding mainly comes from the surrounding soft tissue rather than the socket. After suturing, both sides of the flap are strained and oozing blood is reduced to achieve the purpose of hemostasis. Pachipulusu et al. [20] reported that patients with secondary closure were more comfortable than primary closure because of less postoperative swelling, pain, and trismus. However, despite the closure technique, periodontal healing does not differ in both groups. In the present study, patients with periodontitis and periapical periodontitis tend to be bleeding, and the extraction wound is more susceptible to infection, and suturing was necessary. The present study suggested that surgical extraction of ILTM after flap incision should be closed to prevent infection. However, there are different views on whether a suture is needed for extraction without a flap.

The wound healing of the extracted socket in our study shows that IMTM with periodontitis and periapical periodontitis was the poorest among all groups. Our results didn't report any dry sockets among all groups, and this may be because all wounds closed primarily, which is in line with the previous study [21].

Several studies have shown that approximately 30% of the alveolar ridge is lost due to resorption after tooth extraction. Most bone loss occurs during the first six months after extraction [22, 23]. Computed tomography is the most stable and reliable technology for evaluating bone regeneration, but it is complex, requires more radiation, and has a high follow-up cost. Several studies have demonstrated the utility of OPG in bone regeneration in intra-bony defects using MGV. A previous study evaluated bone healing after a large intra-bony defect using MGV on panoramic radiographs [24]. In the present study, the MGV in periapical periodontitis and periodontitis individuals was significantly lower than in control group. Our finding also shows that elderly individuals tend to have lower MGV than young individuals. On the other hand, a previous study using OPG analysis found that four months after extraction, the rise in MGV was higher in the platelet-rich fibrin group than in the non-platelet-rich fibrin group, indicating accelerated bone regeneration in the study group [25]. Despite OPG being recommended to measure the MGV and bone margin height evaluation not being a standard method, the strength of our study was its homogenous horizontal ILTM that minizines the confiding factors and made patients at a similar level at baseline.

Our finding shows some bone loss regarding the alveolar ridge height and deepening of periodontal pockets distal of the adjacent second molar and for all groups. It was more significant in periodontitis and elderly patient groups, and these findings in line with previous studies [26, 27]. In contrast, Krausz et al. [28] concluded that extraction of ILTM resulted in a considerable gain of alveolar bone height on the distal aspect of the adjacent second molar on the extracted side, whereas slight bone loss was observed on the non-extracted side.

Our study had a strong and convincing rationale with a larger sample size comparing asymptomatic patients with inflammatory conditions associated with ILTM (pericoronitis, pulpits, and periodontists) to control in respect of wound healing, bleeding, and bone density simultaneously. The new clinical findings provided by our results were: (1) In patients with asymptomatic inflammatory conditions like pericoronitis, pulpits, or apical periodontitis, there would be potential bleeding postoperatively, so the surgeon should take place the routine measure to prevent further bleeding. (2) Our results are unique in assessing and comparing wound healing after ILTMs for inflammatory states vs. control subjects. (3) Finally, this study is the first clinical study with a larger sample size that compared bone density at the extraction site between those extraction sites that were associated with inflammation and those control subjects.

Some limitations of this study have to be mentioned, including the inhomogeneous distribution of patients in the groups makes this study prone to bias. The panoramic radiographs are not much accurate in measuring and controlling the cofounder of the MGV. An additional limitation is that we did not assess the duration of the surgery and the lack of a validated post-extraction bleeding scale which would have provided further insights into the subjective perception. In addition, the measurements assessed in the study were not continuous but taken only at specific time points. Moreover, longer follow-up on clinical and radiological parameters is required.

## Conclusion

Inflammatory states associated with ILTM increase the risk of bleeding. Suturing and/or placement of local hemostatic agents over a pressure pack alone may be recommended as the first choice in extractions with localized inflammation, particularly in periapical periodontitis and periodontitis individuals. As a preventative measure, handling hard and delicate tissues with care is recommended to reduce the risk of bleeding. Furthermore, MGV was affected by age and was lower in periapical periodontitis. Although, hemostasis interventions for post-extraction bleeding events do not impact on the MGA.

## Data Availability

All data generated or analyzed during this study are included in this published article.

## Abbreviations

ILTM: Impacted lower third molar
OPG: Orthopantomography radiograph
MGVs: Mean grey values
